# Supplementary material for: A UMLS-based spell checker for natural language processing in vaccine safety
Source: BMC Med Inform Decis Mak. 2007 Feb 12;7:3. doi: 10.1186/1472-6947-7-3 (PMC1805499; doi:10.1186/1472-6947-7-3)
Supplement: Additional file 13 — RAP application directory. Sets up directory for RAP (RDF application for PHP) source code [file 1472-6947-7-3-S13.gz › rap/doc/phpdoc/__filesource/fsource_ontModel__ontModelOntModel.php.html]

xml version="1.0" encoding="iso-8859-1"?


File Source for OntModel.php


# Source for file OntModel.php

Documentation is available at OntModel.php

```
1. <?PHP

3. /**
4. * ----------------------------------------------------------------------------------
5. * Class: OntModel
6. * ----------------------------------------------------------------------------------
7. *
8. * @package     ontModel
9. */


12. /**
13. * Enhanced view of the model that is known to contain ontology data, under a
14. * given ontology vocabulary (such as RDFS). OntModel together with OntClass
15. * and OntResource provide ontology specific methods like addSubClass(),
16. * listSubClasses(), hasSuperProperty(), addDomain() and  listInstances(). This class does not by
17. * itself compute the deductive extension of the graph under the semantic
18. * rules of the language. Instead, we wrap an underlying model with this
19. * ontology interface, that presents a convenience syntax for accessing the
20. * language elements. Depending on the inference capability of the underlying
21. * model, the OntModel will appear to contain more or less triples.
22. * For example, if this class is used to wrap a MemModel or DBModel, only the
23. * relationships asserted by the document will be reported through this
24. * convenience API.
25. * Alternatively, if the OntModel wraps an InfModel (InfModelF / InfModelB),
26. * the inferred triples from the extension will be reported as well.
27. *
28. * <BR><BR>History:
29. * <LI>10-05-2004                : First version of this class.</LI>
30. * <LI>01-17-2005                : bugfix in createOntProperty.</LI>
31. *
32. * @version  V0.9.1
33. * @author Daniel Westphal <mail at d-westphal dot de>
34. *
35. *
36. * @package     ontModel
37. * @access    public
38. ***/
39. class OntModel extends ResModel
40. {
41. /**
42. * Holds a reference to the assoiated vocabulary.
43. * @var        object
44. * @access    private
45. */
46. var $vocabulary;


49. /**
50. * Constructor.
51. * You have to supply a memmodel/dbmodel/infmodel to save the statements and a vocabulary
52. *
53. * @param object Model $model
54. * @access    public
55. */
56. function OntModel(& $model,& $vocabulary)
57. {
58. parent::ResModel($model);
59. $this->vocabulary = & $vocabulary;
60. }

62. /**
63. * Answer a resource that represents a class description node in this model.
64. * If a resource with the given uri exists in the model, it will be re-used.
65. * If not, a new one is created in the updateable sub-model of the ontology model.
66. *
67. * @param    string    $uri
68. * @return    object OntClass
69. * @access    public
70. */
71. function createOntClass($uri = null)
72. {
73. $class = new OntClass($uri);
74. $class->setAssociatedModel($this);
75. $class->setVocabulary($this->vocabulary);
76. $class->setInstanceRdfType($this->vocabulary->ONTCLASS());
77. return $class;
78. }

80. /**
81. * Answer a resource that represents an Individual node in this model.
82. * If a resource with the given uri exists in the model, it will be re-used.
83. * If not, a new one is created in the updateable sub-model of the ontology model.
84. *
85. * @param    string    $uri
86. * @return    object Individual
87. * @access    public
88. */
89. function createIndividual($uri = null)
90. {
91. $individual = new Individual($uri);
92. $individual->setAssociatedModel($this);
93. $individual->setVocabulary($this->vocabulary);
94. return $individual;
95. }

97. /**
98. * Answer a resource that represents an OntProperty node in this model.
99. * If a resource with the given uri exists in the model, it will be re-used.
100. * If not, a new one is created in the updateable sub-model of the ontology model.
101. *
102. * @param    string    $uri
103. * @return    object OntProperty
104. * @access    public
105. */
106. function createOntProperty($uri = null)
107. {
108. $ontProperty = new OntProperty($uri);
109. $ontProperty->setAssociatedModel($this);
110. $ontProperty->setVocabulary($this->vocabulary);
111. $ontProperty->setInstanceRdfType($this->createResource(RDF_NAMESPACE_URI.RDF_PROPERTY));
112. return $ontProperty;
113. }

115. /**
116. * Answer an array that ranges over all of the various forms of class
117. * description resource in this model.
118. * Class descriptions include  domain/range definitions, named  classes and subClass constructs.
119. *
120. * @param    string    $uri
121. * @return    array of object ResResource
122. * @access    public
123. */
124. function listClasses()
125. {
126. //get all statements, with an rdf:type as property
127. $statements= $this->find(null,$this->vocabulary->TYPE(),null);
128. $return = array();
129. $returnIndex=array();
130. foreach ($statements as $statement)
131. {
132. $objectLabel=$statement->getLabelObject();
133. //if it's about a typed Individual
134. if ($objectLabel!=RDF_SCHEMA_URI.RDFS_CLASS)
135. {
136. if (!in_array($objectLabel,$returnIndex))
137. {
138. $returnIndex[]=$objectLabel;
139. $return[]=$statement->getObject();
140. }
141. } else
142. //if it's a "class1 rdf:type rdf:class" construct
143. {
144. $subjectLabel=$statement->getLabelSubject();
145. if (!in_array($subjectLabel,$returnIndex))
146. {
147. $returnIndex[]=$subjectLabel;
148. $return[]=$statement->getSubject();
149. }
150. }
151. }
152. //find all statements about SubClassConstructs
153. $statements= $this->find(null,$this->vocabulary->SUB_CLASS_OF(),null);
154. foreach ($statements as $statement)
155. {
156. //add the statements object to the result
157. $objectLabel=$statement->getLabelObject();
158. if (!in_array($objectLabel,$returnIndex))
159. {
160. $returnIndex[]=$objectLabel;
161. $return[]=$statement->getObject();
162. }
163. }
164. foreach ($statements as $statement)
165. {
166. //add the statements subject to the result
167. $objectLabel=$statement->getLabelSubject();
168. if (!in_array($objectLabel,$returnIndex))
169. {
170. $returnIndex[]=$objectLabel;
171. $return[]=$statement->getSubject();
172. }
173. }
174. return $return;
175. }
176. }
177. ?>
```

Documentation generated on Thu, 7 Jul 2005 13:42:22 +0200 by phpDocumentor 1.3.0RC3
